# Hospitals and Paying Pupils: An Absurd Proposal

**Published:** 1912-05-04

**Authors:** Eva C. E. Lückes

**Affiliations:** Matron of the London Hospital.


### HOSPITALS AND PAYING PUPILS: AN ABSURD PROPOSAL.

**By MISS EVA C. E. LÜCKES, Matron of the London Hospital.**

You have a paragraph about "Hospitals and Paying Pupils" on page 83 of the issue of The Hospital for April 27. I gather from the conclusion of that paragraph that you would like to hear from the "London" on this subject. At the same time, we have no experience of anything on the lines suggested, and you may rest assured that no experiment of so futile a scheme as the one referred to will ever be tried at the London Hospital. I am glad to see that you point out the objections to this absurd proposal very definitely. No one with any knowledge of a hospital ward could imagine it possible that any patients could be victimised by a succession of amateur pupils to be taught at their expense, and anyone who knows how the Sister of a busy ward is occupied would realise at once that the proposal is as impossible to carry out as it is undesirable in itself. One of the chief objects of a Preliminary Training Home is to familiarise the probationers with routine ward duties, *before* they are required to carry them out amongst the patients when they enter the hospital. The number of new probationers—even after they have fulfilled their term of preliminary training—has always to be carefully regulated, to prevent confusion and the difficulties which too many amateur workers together would create in a ward. But to have a series of amateur young women let loose amongst sick people would be so disturbing an element that no well-regulated hospital could sanction such an arrangement.

I fully endorse all you say as to the objections which exist to any such experiment being tried. You will remember, as I do, the harm which resulted in former years from some hospitals making differences in the work and status of paying probationers, and exempting them from the dusting, sweeping, and what may be called the household duties necessary to ensure a proper condition of the patients' immediate surroundings. With such inadequate superficial training, these paying probationers were incompetent ward-managers—if they went on with this sort of semi-training long enough to eventually become Ward Sisters. It was neither fair nor satisfactory from any point of view that they should be allowed to see and do special nursing duties, which obviously ought to be shared by all who are going through a proper training. Nothing of this kind has ever been tolerated at the "London," and when our Training School was first established, towards the end of 1880, one of its fundamental principles was that no differences of any kind could be made in regard to the work, terms, or any part of the training. Social distinctions are ignored, and only the official rank of Sisters, staff nurses, or probationers is recognised.

We have always received paying probationers for periods of not less than three months, with the option of these "three months' engagements" being renewed whenever such an arrangement has appeared mutually desirable. Many probationers have gone through their whole two years' training as paying probationers, because this ensured them freedom to leave at any time when circumstances rendered it necessary for them to do so, and without inflicting the loss on the hospital which occurs when a regular probationer breaks her agreement. All these paying probationers have always taken their proper share in all ward duties, as distinguished from the actual nursing of patients, because we deem it an essential part of the training as a nurse, and also because we can never sanction any distinctions between the duties of one probationer and of another. The recent development, referred to in your last issue, has been in connection with our Preliminary Training Home, where the elementary duties of a probationer are carefully taught by trained Sisters who are appointed for this purpose, and who are able to impart the necessary instruction in the tranquillity of a class-room, apart from patients and from the pressure of work which naturally exists in a ward. The time-table of probationers at a Preliminary Training Home is carefully followed, where there is no high pressure but every opportunity to impress upon beginners the importance of *thoroughness* in carrying out every detail of their future work.

Our new departure has been to allow ladies who are not intending to take up nursing to have, by payment, the advantage of this preliminary work. The fee for paying probationers to enter the hospital for three months has always been £13 13s., *i.e.* at the rate of £1 1s. per week. The charge for a pupil-probationer who is not intending to become a trained nurse is £15 15s. for the seven weeks at our Preliminary Training Home. If the amateur pupils in question wish to complete three months by spending the remaining six weeks in the hospital, the same fee covers this consecutive period of three months' training; but when they are in the wards, just as during the whole time at the Preliminary Training Home, these pupils take their proper share of ward duties, and no distinction of any kind is made between them and the regular probationers. Already some of these special pupil-probationers have returned for another term of three months in the hospital. In every case it is made absolutely clear to them that they must conform to the regulations of the hospital in every respect, if they are to have the advantage of acquiring the knowledge they desire to possess in the atmosphere of the hospital. It is only by this process that anyone can form an idea of what actual nursing and the care of sick people really means. I have always thought that even an elementary knowledge of nursing is invaluable to women in every class of life, and so we have never shut the door to those who wish to learn a little, if circumstances prevent them from becoming fully trained. But three months is the minimum period in which any elementary knowledge of nursing can be gained, and the whole of this brief time must be spent in carrying out *all* elementary duties thoroughly, without shirking any part of the necessary work.

